# Connecting the Dots: Neurobiological Interplay Between Type 2 Diabetes and Alzheimer’s Disease

**DOI:** 10.3390/ijms27073225

**Published:** 2026-04-02

**Authors:** Analía Foncea-Bitrán, Cristián Barros-Osorio, Francisca Arriaza, Catalina Ramírez-López, Lina M. Ruiz, Marlen Barreto, Fernando C. Ortiz, Francisca Cornejo, Gonzalo I. Gómez

**Affiliations:** 1 Center for Integrative Biology, Faculty of Science, Universidad Mayor, Santiago 8580745, Chile; analia.foncea@umayor.com; 2 Institute of Biomedical Sciences, Faculty of Health Sciences, Universidad Autónoma de Chile, Santiago 8581151, Chile; kine.cristianbarros@gmail.com (C.B.-O.); catalina.ramirez11@cloud.uautonoma.cl (C.R.-L.); lina.ruiz@uautonoma.cl (L.M.R.); marlen.barreto@uautonoma.cl (M.B.); 3 Mechanisms of Myelin Formation and Repair Laboratory, Departamento de Biología, Facultad de Química y Biología, Universidad de Santiago de Chile, Santiago 8350347, Chile; franciscarodriguezarriaza@gmail.com (F.A.); fernando.ortiz.c@usach.cl (F.C.O.)

**Keywords:** Alzheimer’s disease, amyloid-β, biomarkers, hyperglycemia, neuroinflammation, cognitive dysfunction, type 2 diabetes mellitus

## Abstract

Diabetes Mellitus is a chronic metabolic disorder characterized by impaired insulin production and/or action, leading to persistent hyperglycemia and insulin resistance. It has been associated with several comorbidities, including cognitive dysfunction, affecting functions such as attention, memory, and processing speed. Mounting evidence indicates a complex relationship between type 2 Diabetes Mellitus (DM2) and neurodegenerative disorders such as mild cognitive impairment and Alzheimer’s disease (AD). Beyond the conventional hallmarks of each pathology, patients with DM2 face an increased risk of neuronal degeneration, while AD is characterized by a marked reduction in insulin receptor density. Although aging, neuroinflammation, and vascular dysfunction have been recognized as key risk factors in AD, the precise molecular mechanisms driving AD pathogenesis remain incompletely understood. Various studies have been conducted to identify reliable biomarkers that elucidate the connection between DM2 and AD, including insulin dysregulation, neuroinflammation, amyloid-β aggregation, and tau hyperphosphorylation. Investigation of these biomarkers is still ongoing, and they may serve not only as diagnostic tools but also as therapeutic targets. Here, we review the current evidence supporting a convergent biological framework between DM2 and AD. Clarifying these shared pathways may improve early detection and guide the development of targeted therapeutic strategies aimed at reducing neurodegeneration in metabolically vulnerable populations.

## 1. Introduction

Diabetes Mellitus (DM) is a metabolic disorder that accounts for 90–95% of all diabetes cases and is considered one of the most significant public health concerns [1]. The global prevalence of DM in 2019 was 9.3% (463 million people), and it is projected to rise to 10.2% (578 million people) by 2030 and 10.9% (700 million people) by 2045 [2]. DM is characterized by disrupted insulin secretion and/or function, leading to persistent hyperglycemia and insulin resistance in peripheral tissues [3,4].

DM comprises two major forms: type 1 diabetes (DM1), characterized by autoimmune destruction of pancreatic β-cells and absolute insulin deficiency, and type 2 diabetes (DM2), which accounts for approximately 90–95% of all diabetes cases and is primarily driven by peripheral insulin resistance and metabolic dysregulation. While both conditions can affect the central nervous system, most epidemiological and mechanistic studies linking diabetes and Alzheimer’s disease (AD) have focused on DM2 due to its strong association with aging, metabolic syndrome, and vascular dysfunction.

Importantly, DM2 is a heterogeneous disorder that encompasses multiple pathophysiological subtypes characterized by different degrees of insulin resistance, impaired insulin secretion, obesity-related metabolic dysfunction, and variable disease progression. Recent classification approaches based on clinical and metabolic parameters have identified distinct diabetes subgroups with differing risks of complications and disease trajectories [5]. In addition, factors such as disease duration, glycemic variability, and recurrent hypoglycemia may differentially influence metabolic and neurological outcomes. These aspects suggest that the relationship between diabetes and neurodegeneration is likely influenced not only by chronic hyperglycemia but also by the underlying metabolic phenotype and progression of the disease.

In addition to its metabolic complications, DM2 has been increasingly associated with cognitive dysfunction, including impairments in executive functions, attention, memory, and processing speed [6,7,8]. Recent studies have identified DM2 as a potential risk factor for cognitive decline, including mild cognitive impairment (MCI), vascular dementia, and particularly AD [7]. Longitudinal cohort and epidemiological studies have reported that about 50-52% of patients with DM2 develop dementia as a late complication, with an increased risk of over 65% for AD [9,10,11]. However, these findings are largely derived from observational studies, and although many analyses adjust for confounding factors such as age, cardiovascular risk, and education level, residual confounding cannot be fully excluded.

Although DM2 has been consistently associated with an increased risk of AD, current evidence does not support a direct causal relationship. Instead, DM2 is increasingly viewed as a condition that modifies and accelerates neurodegenerative processes through interconnected metabolic, vascular, and inflammatory mechanisms.

AD is the most prevalent form of dementia worldwide, affecting nearly 50 million people, and is marked by progressive memory loss and cognitive decline [2]. At the molecular level, AD is characterized by extracellular amyloid-β (Aβ) plaques, intracellular neurofibrillary tangles (NFTs) composed of hyperphosphorylated tau protein, and activation of immune glial cells, mainly microglia [12,13].

Clinical presentations of AD differ between early- and late-onset forms. Early-onset AD is associated with language, visuospatial, or executive function impairments, whereas late-onset AD typically presents with the classic amnestic pattern of the disease [14]. Importantly, comorbidities such as diabetes, cardiovascular dysfunction, and obesity are more frequently observed in late-onset AD, supporting the contribution of metabolic disturbances to disease progression [15,16].

Emerging evidence suggests that DM2 and AD share overlapping pathophysiological mechanisms, particularly alterations in insulin signaling, neuroinflammation, vascular dysfunction, and protein aggregation [9,11].

The relationship between metabolic dysfunction and cognitive impairment has been explored for several decades. Early studies reported that alterations in glucose metabolism and chronic hyperglycemia were associated with learning and memory deficits in both diabetic patients and experimental models [17,18,19]. These observations contributed to the proposal that AD may share key features with metabolic disorders affecting insulin signaling in the brain, leading some authors to describe the condition as a form of “brain diabetes” or “type 3 diabetes” [20]. Although this concept has helped emphasize the role of impaired insulin signaling in neurodegeneration, it remains a debated framework rather than a universally accepted classification of AD.

In AD, insulin receptor density is reduced by 80%, and dysfunctional insulin signaling is believed to play a pivotal role in the development of AD [21]. Therefore, DM2 patients not only have a higher predisposition to cognitive impairments but also an elevated risk of developing AD later in life [22,23]. These cognitive impairments have also been replicated in animal models of diabetes, reinforcing the likelihood of a biological connection [24,25].

Rather than considering DM2 as a direct cause of AD, this review adopts the perspective that DM2 acts as a major disease modifier that accelerates neurodegenerative processes through interconnected metabolic, vascular, and inflammatory mechanisms.

**Figure 1:**
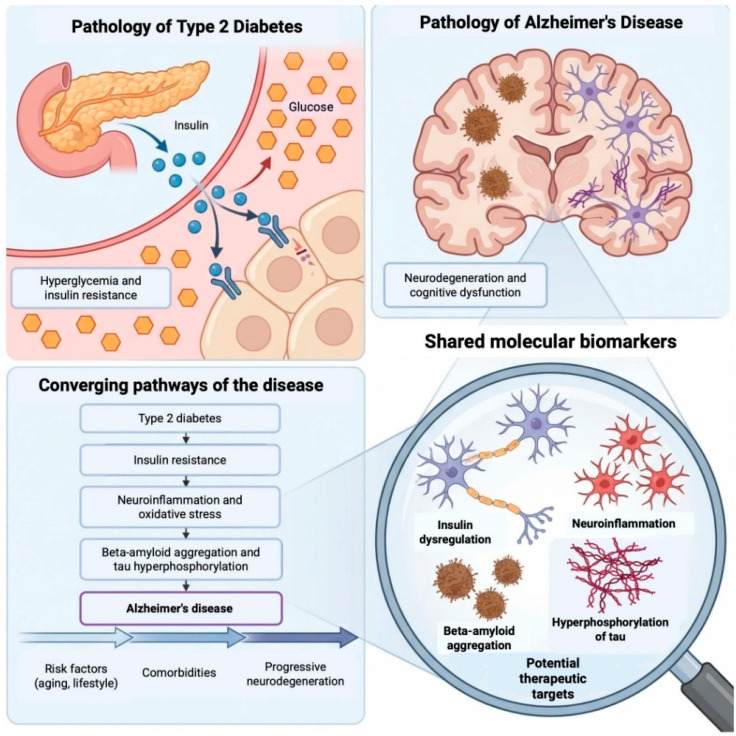
Mechanistic links between type 2 diabetes mellitus (DM2) and Alzheimer’s disease. Chronic hyperglycemia and insulin resistance in DM2 result in altered insulin receptor signaling. This altered insulin signaling compromises neuronal energy homeostasis and synaptic plasticity. Concurrently, hyperglycemia increases oxidative stress through the overproduction of ROS, leading to mitochondrial dysfunction and endothelial damage that contribute to the disruption of the blood–brain barrier. Barrier impairment/permeabilization facilitates infiltration of peripheral immune cells and amplifies central neuroinflammatory responses, mediated by the activation of microglia and astrocytes. Chronic inflammation further promotes the accumulation of Aβ and tau hyperphosphorylation, leading to the formation of neurofibrillary tangles. Together, these pathological processes converge to accelerate neuronal loss, cortical and hippocampal atrophy, and cognitive decline. Created in BioRender. Gomez, G. (2026) https://BioRender.com/jb3sqy9.

This review aims to explore the neurobiological mechanisms linking DM2 to AD, describe the molecular and cellular mechanisms underlying this association, and analyze current advancements in shared biomarkers. By assessing these connections, we aim to provide a comprehensive understanding of the relationship between DM2 and AD from a neurobiological perspective and to suggest targeted therapeutic strategies (Figure 1).

## 2. Physiological Organization of Insulin Signaling in the Brain

### 2.1. Insulin Transport and Brain Entry

Insulin is a peptide hormone secreted by pancreatic β-cells that reaches the central nervous system (CNS) through a regulated transport process across the blood–brain barrier (BBB). This transport allows circulating insulin to access brain parenchyma and interact with insulin receptors expressed in multiple neural cell types [26]. In the brain, insulin functions not only as a metabolic regulator but also as a neurotrophic factor that modulates neuronal survival, synaptic plasticity, and cognitive processes [27,28,29].

### 2.2. Distribution of Insulin Receptors in the Brain

Insulin receptors are widely distributed throughout the brain, with particularly high expression in regions involved in cognitive processing, including the hippocampus, cortex, hypothalamus, olfactory bulb, and pituitary [30,31]. These receptors are predominantly expressed in neurons, although they are also present in glial cells. Their enrichment in hippocampal pyramidal neurons highlights the importance of insulin signaling in learning and memory processes. Functional studies have demonstrated that pharmacological blockade or inhibition of hippocampal insulin receptors leads to impairments in spatial memory and other hippocampal-dependent cognitive tasks, supporting the critical role of insulin signaling in normal brain function [32,33,34].

### 2.3. Insulin Signaling Pathways

Upon insulin binding, the insulin receptor undergoes autophosphorylation and recruits insulin receptor substrates (IRSs), particularly IRS-1. This activation triggers downstream signaling pathways, including phosphoinositide 3-kinase (PI3K), protein kinase B (AKT), and mitogen-activated protein kinase (MAPK). Activation of AKT leads to the inhibition of glycogen synthase kinase 3β (GSK-3β), a kinase involved in multiple cellular processes including cytoskeletal regulation and protein phosphorylation [35,36,37].

### 2.4. Neurotrophic and Metabolic Roles of Insulin

In addition to its role in glucose metabolism, insulin regulates several cellular processes that are essential for neuronal function. These include protein synthesis, post-translational modifications, cytoskeletal organization, and the maintenance of axonal integrity [28,38]. Through these mechanisms, insulin signaling contributes to neuronal survival, synaptic stability, and the maintenance of neural network function.

## 3. Pathophysiological Alterations Linking DM2 and Alzheimer’s Disease

DM2 can impact brain function through various mechanisms, including glucose-induced toxicity, BBB disruption, increased production of reactive oxygen species (ROS), and insulin resistance in the brain [39,40]. Hyperglycemia and reduced brain glucose uptake also contribute to the formation of advanced glycation end products (AGEs), which are now considered a molecular bridge between DM2 and AD pathologies [39,41,42].

Increasing evidence indicates that the brains of AD patients exhibit molecular features resembling insulin-deficient states, including reduced insulin and insulin-like growth factor (IGF) signaling, together with elevated oxidative and inflammatory stress markers [43,44,45,46]. These observations have led to the hypothesis that insulin dysfunction contributes directly to neurodegenerative processes, supporting the idea that DM2 and AD share common pathogenic pathways. The principal molecular mechanisms linking metabolic dysfunction to neurodegeneration are summarized in Table 1.

### 3.1. Brain Insulin Resistance

Brain insulin resistance has emerged as a central mechanism linking DM2 with neurodegeneration. Insulin plays a fundamental role in brain function, particularly in cognition and memory. Beyond its well-known role in regulating glucose metabolism, insulin signaling regulates neuronal survival, synaptic plasticity, and inflammatory responses [60]. Dysregulation of these pathways may contribute to neurodegenerative processes associated with AD.

At the molecular level, brain insulin resistance is frequently associated with abnormal serine phosphorylation of IRS-1. Under physiological conditions, IRS-1 transduces insulin receptor signaling through tyrosine phosphorylation, enabling activation of the PI3K-AKT pathway and downstream metabolic and neurotrophic responses [61]. In contrast, phosphorylation of IRS-1 at specific serine residues disrupts its interaction with the insulin receptor and inhibits downstream signaling, representing a key molecular feature of insulin resistance in DM2 [62]. Stress-activated kinases such as JNK, which are activated by inflammatory cytokines, oxidative stress, and metabolic overload, can promote this inhibitory phosphorylation of IRS-1 [63]. Importantly, increased IRS-1 serine phosphorylation has also been detected in the hippocampus and cortex of AD patients and experimental models, where it correlates with impaired insulin signaling and cognitive decline [64,65]. Moreover, Aβ oligomers can activate JNK signaling in neurons, further enhancing IRS-1 inhibitory phosphorylation and contributing to a state of brain insulin resistance [64,66]. Together, these findings suggest that stress-kinase-mediated IRS-1 dysfunction represents a key molecular mechanism linking metabolic dysregulation in diabetes with brain insulin resistance and neurodegeneration, supporting the concept that metabolic stress-induced signaling alterations act as mechanistic drivers of brain insulin resistance within the modifier framework linking DM2 and AD.

The mammalian target of rapamycin (mTOR) pathway represents another key molecular link between metabolic dysfunction and neurodegeneration. mTOR acts as a central nutrient and insulin sensor downstream of the PI3K-AKT pathway, coordinating protein synthesis, cellular growth, and autophagy. In DM2, chronic mTORC1 activation driven by overnutrition and hyperinsulinemia promotes insulin resistance by inhibiting IRS-1 via S6K-mediated phosphorylation, further impairing insulin signaling [67]. Importantly, dysregulated mTOR signaling has also been reported in AD, where excessive mTOR activity suppresses autophagy–lysosomal degradation pathways and favors the accumulation of misfolded proteins such as Aβ and hyperphosphorylated tau [68]. In addition, abnormal mTOR activation contributes to mitochondrial dysfunction, oxidative stress, and synaptic impairment. Together, these findings position mTOR signaling as a key molecular bridge linking metabolic disturbances in diabetes with protein aggregation and neurodegenerative processes characteristic of AD.

Another mechanism linking metabolic dysfunction with AD pathology involves insulin-degrading enzyme (IDE), a protease responsible for the degradation of both insulin and Aβ. IDE plays a central role in insulin clearance, and reduced IDE activity has been associated with hyperinsulinemia and glucose intolerance. In conditions such as DM2, chronic hyperinsulinemia may increase competition for IDE, limiting its capacity to degrade Aβ and thereby favoring amyloid accumulation [69]. Experimental studies have shown that reduced IDE activity results in both elevated insulin levels and increased Aβ deposition, supporting a mechanistic link between metabolic dysfunction and amyloid pathology [70]. Although the extent of this competitive interaction in the brain remains under debate, the IDE pathway provides an important conceptual bridge between peripheral insulin resistance and neurodegenerative processes in AD. Therefore, insulin levels can also serve as a surrogate marker for the efficiency of Aβ clearance, acting as an indirect indicator of Alzheimer’s-related pathological processes [60,71]. In this sense, brain insulin resistance or impaired insulin signaling may be key factors linking metabolic dysfunction characteristic of DM2 with the neurodegenerative changes observed in AD.

### 3.2. Glucose Metabolism and BBB Dysfunction

Hyperglycemia and impaired glucose metabolism represent key pathological features linking DM2 and AD. Chronic metabolic dysregulation alters the activity of glucose transporters such as GLUT1 and GLUT3 and disrupts insulin-dependent signaling pathways including PI3K and MAPK [72,73].

Structural and functional integrity of the BBB is frequently compromised in DM2 due to chronic hyperglycemia, oxidative stress, and systemic inflammation [74,75]. In fact, hyperglycemia in DM2 can affect GLUT transporter activity, compromise the integrity of the BBB and promote the entry of neurotoxic substances into the brain, exacerbating inflammation and neuronal damage [76,77].

Importantly, these alterations should be considered within the broader context of neurovascular unit dysfunction. In DM2, chronic metabolic stress promotes endothelial dysfunction, pericyte loss, microvascular damage, and reduced cerebral blood flow, thereby compromising the structural and functional integrity of the BBB [78,79,80]. This vascular dysfunction disrupts nutrient delivery, impairs the clearance of metabolic waste products such as Aβ, and promotes neuroinflammation and neuronal injury. Increasing evidence therefore suggests that the relationship between DM2 and AD is not only mediated by metabolic and neuronal mechanisms but also involves a significant vascular component, in which microvascular pathology and BBB impairment contribute to neurodegenerative processes.

These metabolic and vascular alterations contribute to cognitive dysfunctions, highlighting the importance of glycemic control in AD prevention [81,82].

### 3.3. Mitochondrial Dysfunction

**Figure 2:**
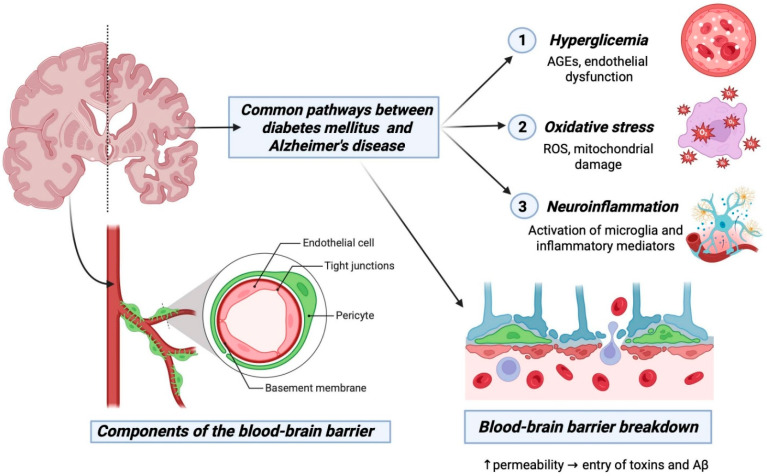
Blood–Brain Barrier Dysfunction in Diabetes Mellitus and Alzheimer’s Disease: Mechanisms and Therapeutic Targets. This figure illustrates the intricate relationship between DM2 and AD through the lens of BBB dysfunction. The BBB, composed of endothelial cells, astrocytes, neurons, and pericytes, acts as a critical defense mechanism for the brain against neurotoxic compounds. The figure outlines the breakdown of the BBB in AD, emphasizing the role of impaired tight junctions and adherents’ junctions in brain microvascular endothelial cells. Furthermore, the figure delves into the similarities between DM2 and neurodegenerative diseases, such as vascular dementia and AD, showcasing how hyperglycemia, oxidative stress, and chronic inflammation contribute to BBB impairment in DM2. Overall, this comprehensive figure provides insights into the multifaceted mechanisms of BBB dysfunction in the context of DM2 and AD, offering potential avenues for therapeutic interventions. Created in BioRender. Barros, C. (2026) https://BioRender.com/nu97kgx.

Mitochondrial dysfunction is another key mechanism connecting metabolic disorders and neurodegeneration. Impaired insulin signaling and chronic hyperglycemia disrupt mitochondrial bioenergetics, increase ROS production, and reduce neuronal resilience to metabolic stress. In this context, vascular factors such as vascular endothelial growth factor (VEGF), a key regulator of angiogenesis and neurovascular function, have been implicated in modulating neuronal survival. Experimental studies have shown that VEGF can mitigate Aβ-induced mitochondrial depolarization and promote mitochondrial biogenesis, thereby improving cognitive performance [83]. Although VEGF did not significantly affect autophagy or mitophagy, its role in preserving mitochondrial function highlights a potential link between vascular dysfunction and neuronal metabolism in the context of DM2 and AD [83,84,85,86] (Figure 2).

### 3.4. Amyloid and Tau Pathology

Insulin plays an important role in regulating the metabolism of Aβ and tau, the two principal pathological proteins that form amyloid plaques and NFTs, respectively [46,87,88,89]. Reduced insulin production, as observed during aging and AD, correlates with decreased insulin activity in brain regions involved in cognition, including the frontal cortex, hippocampus, and hypothalamus [90].

One of the major downstream consequences of disrupted insulin signaling is the accumulation of amyloid-β and tau pathology. Insulin resistance in the brain has been associated with atrophy and AD phenotype, including tau pathology and Aβ accumulation [91]. Insulin is vital not only for glucose uptake but also for maintaining axonal integrity through its regulation of cytoskeletal proteins and growth factors. The reduction in insulin production interferes with these processes, thereby promoting synaptic dysfunction and neurodegeneration [92].

Soluble oligomers of the Aβ peptide, widely distributed in the brain, have been implicated in pathologies like AD, inducing hyperphosphorylation of neuronal tau protein, oxidative stress, neurodegeneration, synaptic loss, and inhibition of synaptic plasticity [93,94,95,96]. These features are also observed in insulin-involved AD pathogenesis in patients with DM2 [97]. Moreover, disrupted insulin pathways contribute to the accumulation of Aβ [98], suggesting that impaired insulin signaling may underlie a shared pathogenic mechanism (Figure 2).

On the other hand, a negative correlation has been observed between insulin signaling and tau phosphorylation [99]. When insulin signaling is impaired, GSK-3β remains active, resulting in tau hyperphosphorylation and NFT formation [100,101]. Therefore, the interplay between metabolic dysfunction and neurodegenerative processes not only supports the hypothesis that brain insulin resistance is a common factor but also highlights potential therapeutic avenues aimed at restoring insulin signaling to slow disease progression [102].

### 3.5. Genetic Susceptibility

Genetic factors further modulate the link between DM2 and AD. The ε4 allele of apolipoprotein E (APOE), which is the strongest genetic risk factor associated with late-onset AD, is also linked to impaired lipid and glucose metabolism. The APOE ε4 allele particularly influences the development and progression of late-onset AD, highlighting its critical role in the neurobiological interplay between metabolic dysregulation and neurodegeneration in the aging population [103]. Individuals carrying the ε4 allele exhibit increased risk of hyperglycemia, insulin resistance, and Aβ aggregation [103,104,105]. By contrast, APOE ε2 confers protection for cognitive impairments and insulin resistance, while ε3 is considered neutral [105,106,107,108].

**Figure 3:**
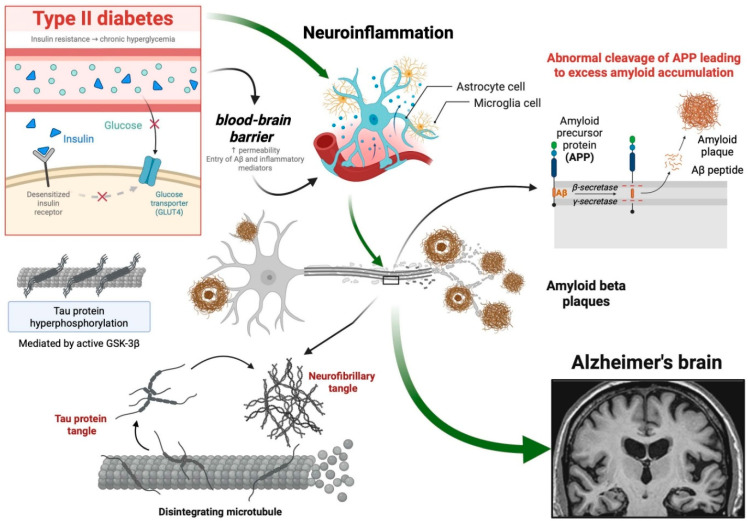
Interconnected Mechanisms Linking Diabetes Mellitus Type 2 and Alzheimer’s Disease. The figure illustrates the interconnected mechanisms that establish a link between DM2 and AD. It focuses on shared elements such as insulin resistance, chronic inflammation, and common brain proteins. The impact of insulin on cognitive processes, Aβ peptide levels, and tau protein hyperphosphorylation is highlighted. Additionally, it shows mechanisms like neuroinflammation, Aβ accumulation, and tau phosphorylation, emphasizing blood biomarkers for AD diagnosis. In the amyloidogenic pathway, amyloid precursor protein is first cleaved by β-secretase and subsequently cleaved by γ-secretase to generate Aβ peptides. The accumulation and aggregation of Aβ peptides results in neurotoxic amyloid plaques. This figure provides a visually comprehensive overview of the complex interplay between DM2 and AD, unraveling the interconnected mechanisms that contribute to their shared pathophysiology and mutual promotion of cognitive dysfunction. Created in BioRender. Gomez, G. (2026) https://BioRender.com/19y3vbh.

The overlapping role of APOE in lipid transport, neuronal repair, and glucose metabolism suggests that it is a key integrator of vascular, metabolic, and neurodegenerative processes [103]. Studies have found that APOE ε4 carriers show specific cognitive deficits, particularly in long-term memory, while individuals with diabetes are more prone to working memory impairments [109]. Importantly, the co-occurrence of APOE ε4 and diabetes does not appear to synergistically elevate AD risk, suggesting distinct but converging mechanisms of damage [109] (Figure 3).

Emerging evidence suggests that the APOE ε4 allele may also influence metabolic and insulin signaling pathways in the brain. APOE4 has been associated with impaired cerebral glucose metabolism, reduced neuronal glucose uptake, and alterations in insulin receptor signaling in key brain regions involved in cognition [110,111,112]. In addition, APOE4 has been linked to BBB dysfunction and vascular alterations that may further compromise nutrient delivery and brain metabolic homeostasis [113,114]. These metabolic disturbances may contribute to the development of brain insulin resistance, thereby reinforcing the pathogenic overlap between diabetes and AD. In this context, APOE4 may act as a genetic modifier that exacerbates the metabolic vulnerability of the brain, potentially amplifying the impact of systemic metabolic disorders such as DM2 on neurodegenerative processes.

These findings position APOE4 not only as a genetic risk factor but also as a modulator of metabolic vulnerability, reinforcing the interaction between genetic and metabolic pathways in AD pathogenesis.

## 4. Glial-Dependent Neuroinflammatory Mechanisms as Possible Contributors to Diabetes Mellitus Pathophysiology

Neuroinflammation is a hallmark of several neurodegenerative diseases, including AD. It constitutes a complex innate immune response against harming agents, aiming to resolve the triggering threat to restore the homeostasis of the CNS. Microglia and astrocytes—the main glial cells in the CNS—are key actors in this process, as they reduce infection and eliminate cell debris, pathogens, and misfolded proteins. In physiological conditions, microglia, the resident immune cells of the CNS, maintain tissue homeostasis through surveillance, synaptic remodeling, and debris clearance. However, under neuroinflammation, they become dysregulated, leading to sustained damage and neuronal dysfunction [115,116,117]. In this scenario, glial cells experience morphological, molecular and functional changes referred to as “reactive gliosis”, thereby promoting persistent neuroinflammation [115,116]. This maladaptive response is driven by interconnected mechanisms, including activation of the NLRP3 inflammasome, engagement of the advanced glycation end-products (AGEs)–receptor for AGEs (RAGE) axis, and metabolic reprogramming, all of which shift microglial phenotype to harmful forms that will in turn activate astrocytes, amplifying damage signaling [115,118,119,120]

In the past few decades, microglial activation has been characterized in the M1/M2 framework; however, recent consensus recommends avoiding this dichotomic definition to describe the different microglial states [115,121]. Microglia plasticity shows several stages associated with more detrimental or pro-inflammatory effects versus protective phenotypes. Then, stimuli such as interferon-γ or damage-associated signals lead to the production of microglia cytokines (e.g., TNF-α, IL-1β, IL-6), reactive oxygen species (ROS), and nitric oxide [115,121]. Persistent microglia activation in detrimental pro-inflammatory stages creates a neurotoxic environment that promotes neuronal injury [116]. In contrast, protective phenotypes support tissue repair and resolution of inflammation through anti-inflammatory cytokines such as IL-10 and TGF-β [122,123,124]. In chronic conditions like diabetes, this balance is disrupted, favoring a sustained pro-inflammatory phenotype [125,126].

A key mediator of microglial-driven inflammation is the NLRP3 inflammasome. Its activation requires an early priming step mediated by a signal downstream of pattern recognition receptors such as TLRs or RAGE, such as NF-κB, followed by a second activation signal triggered by mitochondrial dysfunction, ROS, or ionic imbalance [127,128]. Recent evidence indicates that during neuroinflammation, the assembly of the NLRP3 complex in microglia activates caspase-1, leading to pyroptosis [118,129]. Since chronic hyperglycemia promotes mitochondrial dysfunction and oxidative stress, in diabetes mellitus, facilitation of both priming and activation of the inflammasome is expected [130].

Another possible mechanism involved is the AGEs–RAGE pathway, which represents a key link between metabolic dysregulation and neuroinflammation [131]. Hyperglycemia drives the formation of AGEs, which accumulate in tissues and bind to RAGE expressed on microglia [132]. This AGE-RAGE interaction activates NF-κB along with other downstream inflammatory pathways, increasing cytokine production and ROS generation [133,134]. Importantly, RAGE signaling establishes a feed-forward loop, as NF-κB upregulates RAGE expression, amplifying microglial responsiveness [135,136,137,138,139,140]. Additionally, AGEs and other RAGE ligands contribute to mitochondrial dysfunction, further enhancing inflammasome activation.

Metabolic reprogramming is another critical determinant of microglial function. Pro-inflammatory microglia undergo a shift from oxidative phosphorylation to aerobic glycolysis, supporting rapid energy demands and biosynthesis of inflammatory mediators [115]. Evidence indicates that this shift is triggered by the accumulation of metabolites such as succinate and others that, in turn, stabilize HIF-1α and promote IL-1β expression [119,141,142] (Kelly & O’ Neill, 2015; Miao et al., 2023; Tannahill et al., 2013). In contrast, protective or anti-inflammatory phenotypes of microglia rely on mitochondrial oxidative metabolism and fatty acid oxidation [119]. In diabetes, patients face chronic nutrient excess and insulin resistance, leading to mitochondrial dysfunction that might induce a glycolytic metabolism, promoting the reprogramming of microglia toward harmful and pro-inflammatory forms. In this scenario, dysregulation of key metabolic regulators such as AMPK and mTOR further enhances NLRP3 activation and cytokine production [143].

In summary, neuroinflammatory mechanisms act collectively as an integrated self-amplifying network where AGEs–RAGE signaling primes inflammatory pathways, metabolic reprogramming enhances ROS production, and NLRP3 activation amplifies cytokine release. The latter might certainly foster microglial polarization shifts to detrimental stages that will further promote neuroinflammation by activating astrocytes and releasing pro-inflammatory factors [121]. In diabetes mellitus, systemic metabolic dysfunction trigger CNS inflammation, contributing to the reactive gliosis, neuronal damage and cognitive decline that characterize neurodegenerative diseases such as AD.

## 5. Biomarkers Linking Diabetes and Alzheimer’s Disease

In recent years, the development of highly sensitive immunoassays has significantly advanced the early and less invasive detection of AD biomarkers in plasma and cerebrospinal fluid (CSF) [144]. Given the growing evidence that DM2 contributes to several pathogenic mechanisms described above, including insulin resistance, metabolic dysfunction, and neuroinflammation, neurodegeneration has been proposed as a key biological link between DM2 and AD [145].

### 5.1. Classical AD Biomarkers

The neuropathological hallmarks of AD include extracellular Aβ plaques and intracellular NFTs composed of hyperphosphorylated tau protein [53]. In CSF, lower levels of Aβ42 and lower Aβ42:Aβ40 ratios are commonly observed in AD [52]. This decrease reflects the sequestration of Aβ42 into amyloid plaques in the brain, which is accompanied by increased levels of total tau (t-tau) and phosphorylated tau at Thr181 (p-tau181) in CSF [146]. Additionally, increases in t-tau and p-tau181 in CSF tend to correlate with Aβ burden rather than with NFT load [147], suggesting that they may reflect early pathophysiological changes rather than late-stage neuronal loss [54].

### 5.2. Plasma Biomarkers

Recent advances in plasma biomarkers have demonstrated that phosphorylated tau species, particularly p-tau181, p-tau217, and p-tau231, can predict AD pathology with high accuracy [148]. Among these, plasma p-tau217 has shown particularly strong diagnostic performance in distinguishing AD patients from cognitively normal individuals, with high concordance with amyloid PET and tau PET imaging across multiple cohort studies [148,149,150]. In addition, p-tau231 has been proposed as an early plasma biomarker associated with Aβ pathology, showing strong correlation with amyloid PET positivity in preclinical and early-stage AD [151].

Tau hyperphosphorylation remains a central pathological mechanism in AD and may be exacerbated by chronic hyperglycemia, as observed in diabetes [152]. Elevated glucose levels promote tau hyperphosphorylation in hippocampal neurons, facilitating its detachment from microtubules, misfolding, and aggregation into NFTs, inducing cognitive dysfunction in diabetes [153,154,155,156]. This pathological process disrupts axonal transport and cytoskeletal integrity, resulting in neuronal dysfunction, synaptic damage, and cell death [154]. In hyperglycemic conditions, tau is also subject to proteolytic cleavage by endogenous enzymes, such as caspases and calpains, which enhances its aggregation propensity [55,157]. These cleaved forms of tau further promote NFT formation and correlate with cognitive decline [59,158].

### 5.3. Metabolic Biomarkers

Metabolic dysregulation associated with DM2 may also influence biomarkers related to AD pathology.

C-peptide, traditionally considered a byproduct of insulin synthesis, has recently been recognized as a bioactive molecule capable of modulating insulin signaling [51]. Emerging evidence suggests that pathways associated with C-peptide signaling may influence metabolic and neurodegenerative processes relevant to DM2-associated cognitive decline [57,58].

Hyperglycemia also exacerbates cognitive impairment through osmotic stress, oxidative damage, and inflammatory responses. The formation of AGEs promotes ROS production and the release of inflammatory cytokines such as IL-1β and IL-6, which can be detected as systemic inflammatory biomarkers associated with neurodegenerative processes [56,159].

However, despite these advances, several limitations should be considered when interpreting the role of metabolic biomarkers in AD. First, many diabetes-related biomarkers primarily reflect systemic metabolic status rather than brain-specific pathological processes, which limits their specificity for AD and their direct association with core neuropathological features such as Aβ deposition and tau pathology. Second, although epidemiological studies consistently report an association between diabetes and increased risk of AD, this relationship remains largely correlational, which restricts the predictive value of metabolic biomarkers when considered in isolation.

A major challenge in this field is the lack of reliable methods to directly assess brain insulin resistance, a key mechanistic link proposed to connect metabolic dysfunction with neurodegeneration. While peripheral insulin resistance can be readily evaluated using clinical measures, its central counterpart remains difficult to quantify in vivo, limiting the translation of this concept into clinically actionable biomarkers.

These limitations are consistent with a multifactorial model of AD, in which metabolic dysfunction represents a contributing but non-sufficient component of disease pathogenesis. In this context, emerging approaches increasingly focus on integrative biomarker strategies, combining metabolic indicators with AD-specific biomarkers (such as plasma or CSF tau species), genetic risk factors (including APOE genotype), and neuroimaging data. Such multi-layered models may improve disease stratification and predictive accuracy by capturing the complex interplay between systemic metabolism and brain-specific pathology, thereby offering a more comprehensive framework for understanding and diagnosing AD.

## 6. Therapeutic Strategies Targeting Metabolic Dysfunction

The shared mechanisms described above, including insulin resistance, mitochondrial dysfunction, chronic inflammation, and metabolic dysregulation, have prompted increasing interest in repurposing anti-diabetic drugs as potential therapeutic strategies for cognitive decline and AD, particularly in patients with DM2.

### 6.1. Insulin-Based Therapies

Given the central role of insulin signaling in brain physiology, therapeutic strategies aimed at restoring insulin activity in the CNS have attracted considerable attention. Experimental evidence suggests that insulin may protect against Aβ synaptotoxicity by promoting Aβ elimination through the regulation of lipid metabolism, proteases, and IDE [50]. In AD, impaired insulin signaling has been associated with reduced insulin receptor activity, decreased Aβ clearance, and increased tau phosphorylation, leading to synaptic dysfunction, neuroinflammation, and neuronal loss [160,161,162]. Postmortem studies of AD patients have revealed deficient insulin signaling in regions associated with cognition, such as the frontal cortex and hippocampus [163]. These abnormalities are associated with both Aβ accumulation and tau hyperphosphorylation, underscoring the relevance of insulin-related pathways in AD pathogenesis [164]. Restoring insulin signaling may therefore represent a promising strategy to counteract neurodegenerative processes.

Emerging therapeutic strategies now aim to restore brain insulin function. Intranasal insulin administration has shown promise by enhancing cognitive performance in both preclinical and clinical studies [164]. This approach increases brain insulin availability without affecting blood glucose, reducing the risk of hypoglycemia.

Other therapeutic targets include insulin-like growth factor-1 (IGF-1) and insulin receptor substrate-1 (IRS-1), which are frequently impaired in AD brains [64,163].

### 6.2. Insulin Sensitizers

Among insulin-sensitizing agents, thiazolidinediones, such as pioglitazone, have attracted attention for their anti-inflammatory and metabolic effects. Activation of PPARγ pathways can reduce systemic inflammation, improve endothelial function, and modulate metabolic pathways associated with neurodegeneration. Experimental studies suggest that pioglitazone may exert vasculoprotective and neuroprotective effects, including the reduction of inflammatory mediators and improvement of vascular function [165].

Clinically, in patients with DM2, pioglitazone has been associated with improved glycemic control and decreased inflammatory mediators such as C-reactive protein and VEGF. However, potential adverse effects, including peripheral edema, weight gain, and hypoglycemia when combined with insulin or other hypoglycemic agents, must also be considered [165].

However, despite promising preclinical findings, large clinical trials evaluating thiazolidinediones, including rosiglitazone and pioglitazone, have failed to demonstrate consistent cognitive benefits in patients with AD [166,167]. These limitations have been attributed to factors such as insufficient CNS penetration, variability in patient populations, and differences in disease stage at the time of intervention.

### 6.3. Glucose-Lowering Therapies

Incretin-based therapies have also emerged as promising candidates for the treatment of metabolic and neurodegenerative disorders. Emerging evidence suggests that treatment with glucagon-like peptide-1 receptor agonists (GLP-1RAs) may offer therapeutic benefits by improving insulin signaling and reducing the incidence of dementia in patients with DM2 [57,58]. In addition to their glucose-lowering effects, GLP-1RAs have been shown to exert anti-inflammatory and neuroprotective actions in the CNS.

Another promising therapeutic strategy involves sodium-glucose cotransporter 2 (SGLT2) inhibitors, which lower blood glucose by inhibiting renal glucose reabsorption. Beyond their metabolic effects, emerging evidence suggests that these drugs may influence neuroinflammatory pathways and brain energy metabolism [165,168,169]. Population-based and observational studies have compared the effects of SGLT2 inhibitors with other anti-diabetic drugs, such as dipeptidyl peptidase-4 (DPP4) inhibitors, indicating that SGLT2 inhibitors may be more effective in reducing the risk of dementia in DM2 patients [170,171]. The neuroprotective effects of these drugs extend beyond their anti-diabetic properties, suggesting a direct impact on brain health and cognitive function [172]. Furthermore, SGLT2 inhibitors have been shown to prevent memory impairment in AD animal models, showing beneficial effects on neurogenesis, synaptic plasticity, and neurodegeneration [173]. These findings suggest that targeting glucose metabolism through SGLT2 inhibition may offer novel therapeutic strategies for managing cognitive decline associated with AD [170].

While glycemic control remains the primary goal in the management of diabetes, certain therapeutic strategies may inadvertently increase the risk of hypoglycemia, a factor associated with cognitive impairment and memory deficits. Importantly, meta-analyses of observational studies have shown that severe hypoglycemia, typically defined as episodes requiring external assistance, has been consistently linked to an increased risk of cognitive decline and dementia [174,175], whereas the effects of mild or moderate hypoglycemia remain less clear and may depend on frequency and patient vulnerability. Ramirez-Rincón et al. emphasized the importance of individualized diabetes treatment strategies that consider patient-specific factors such as cardiovascular risk, comorbidities, and potential adverse events, including hypoglycemia [176]. Overly aggressive glucose-lowering regimens, particularly in vulnerable populations, may increase hypoglycemia risk and potentially accelerate cognitive decline.

Conversely, maintaining optimal glycemic control through careful management may help mitigate neurodegenerative processes associated with AD, if hypoglycemia is avoided [177,178,179]. These observations highlight the need for balanced metabolic management strategies that aim not only to control blood glucose levels but also to minimize hypoglycemic events to preserve cognitive function.

Despite these promising findings, several limitations should be considered when interpreting the potential neuroprotective effects of antidiabetic drugs. Much of the current evidence derives from observational studies or experimental models, whereas randomized clinical trials specifically designed to evaluate cognitive outcomes remain limited. Observational studies may also be affected by confounding factors, including differences in metabolic control, cardiovascular risk profiles, and healthcare access among treated populations. In addition, the extent to which many antidiabetic agents directly affect the CNS remains uncertain, as the ability of these drugs to cross the BBB varies. Therefore, further clinical studies are required to determine whether the observed associations reflect direct neuroprotective effects or indirect benefits mediated through improved systemic metabolic control.

Also, despite strong mechanistic rationale and encouraging preclinical data, the translation of diabetes-related therapies into effective treatments for AD has yielded mixed and often disappointing results. Several clinical trials targeting insulin signaling and metabolic pathways have failed to demonstrate consistent cognitive benefits in AD patients.

Importantly, these findings do not necessarily contradict the role of metabolic dysfunction in AD but rather support the view that metabolic alterations act as disease modifiers rather than primary drivers of neurodegeneration. In this context, targeting metabolic pathways alone may be insufficient to halt disease progression, particularly in established stages of AD where multiple pathological processes coexist.

For example, intranasal insulin has shown variable outcomes across studies, with some trials reporting modest cognitive improvements while others failed to replicate these effects, potentially due to differences in dosing, patient stratification, and disease stage [180,181,182]. Similarly, GLP-1RAs, such as liraglutide and semaglutide, have demonstrated neuroprotective effects in experimental models, but clinical evidence in AD remains limited and inconclusive [183,184,185].

Metformin, one of the most widely used antidiabetic drugs, has also been investigated for its potential neuroprotective effects due to its ability to modulate AMPK signaling, reduce oxidative stress, and influence metabolic homeostasis [186,187]. However, clinical evidence regarding its impact on cognitive decline and AD progression remains inconsistent, with some studies suggesting potential benefits while others report neutral or even adverse cognitive outcomes [188,189]. These discrepancies may reflect differences in treatment duration, patient metabolic status, and disease stage.

Taken together, these translational limitations are consistent with a multifactorial model of AD, in which metabolic dysfunction contributes to disease progression but is unlikely to represent a standalone therapeutic target.

## 7. Integrating Insulin Signaling, Metabolism, and Genetic Susceptibility in AD

We propose a model in which metabolic dysfunction, vascular impairment, and genetic susceptibility converge to create a state of increased brain vulnerability, in which DM2 accelerates the onset and progression of AD pathology. This perspective distinguishes between a direct causal model, in which DM2 would independently trigger AD pathology, and a modifier model, in which metabolic dysfunction amplifies pre-existing vulnerability driven by aging, genetic factors, and other pathological processes.

Importantly, this conceptual framework is consistent with the current translational evidence. The limited and often inconsistent outcomes observed in clinical trials targeting metabolic pathways in AD do not negate the role of metabolic dysfunction but rather support the notion that these pathways act as modulators within a multifactorial disease context. Interventions aimed exclusively at correcting metabolic alterations may therefore be insufficient to produce significant clinical benefit, particularly in established stages of the disease where neurodegeneration is already driven by multiple converging mechanisms.

Metabolic dysfunction associated with DM2 has been increasingly recognized as a contributor to neurodegenerative processes linked to AD. Insulin resistance, hyperglycemia, and dyslipidemia can affect brain structure and function [190,191,192], and have been associated with brain atrophy and cognitive impairment even in individuals without dementia [193,194]. In addition, much of the epidemiological evidence linking DM2 and AD is derived from observational studies, which, despite adjusting for major confounding factors, cannot fully exclude residual confounding and therefore limit causal interpretation.

While the concept of “type 3 diabetes” has been proposed to highlight the role of insulin resistance in the brain, AD is a multifactorial disorder involving metabolic, vascular, genetic, and inflammatory mechanisms.

Neuroimaging studies further support this metabolic connection. For example, positron emission tomography using fluorodeoxyglucose (FDG-PET) has revealed reduced glucose uptake in cognitively normal individuals carrying the APOE ε4 allele, particularly in the temporal lobe [195].

The APOE ε4 allele is the strongest genetic risk factor for late-onset AD and has been associated with accelerated cognitive decline [196,197]. In contrast, APOE ε3, the most common isoform, is generally considered neutral with respect to disease risk [196,198].

Interestingly, some studies suggest that APOE ε4 carriers with diabetes may experience distinct cognitive trajectories compared to non-carriers. While APOE ε4 primarily affects long-term memory, diabetes is more closely linked to deficits in working memory [109]. This suggests that DM2 and APOE ε4 may influence cognition through partially independent but converging mechanisms.

**Figure 4:**
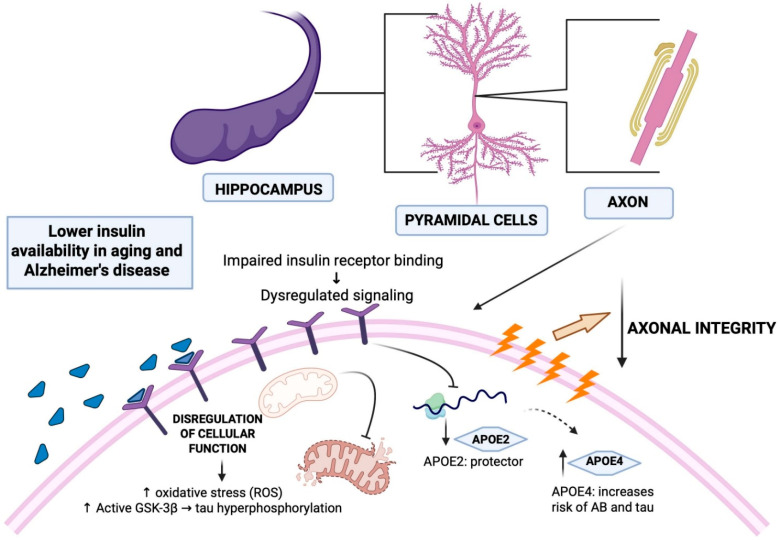
Interplay of Insulin, APOE Genotypes, and Alzheimer’s Disease Onset. This figure illustrates the intricate relationships between insulin, APOE genotypes, and AD. Insulin, a crucial trophic factor in the CNS, is transported across the BBB and interacts with insulin receptors, which are widely expressed in the hippocampus. The figure highlights how alterations in insulin levels and signaling contribute to neurodegeneration and cognitive impairment in the context of DM2 and aging. It visualizes how alterations in insulin levels and signaling, particularly in brain regions like the hippocampus, contribute to neurodegeneration in the context of diabetes mellitus and aging. The figure also highlights the impact of APOE genotypes on AD risk, emphasizing the neuroprotective role of APOE2, and the increased risk associated with APOE4. Created in BioRender. Barros, C. (2026) https://BioRender.com/apr43xk.

The importance of considering APOE genotype and diabetes as potential risk factors for AD highlights the need for further research to elucidate how these factors contribute to the development and progression of the disease [109,199] (Figure 4). Insights gained from such studies could pave the way for targeted interventions and personalized treatment approaches in the field of AD research and management.

Together, these findings suggest that metabolic dysfunction and genetic susceptibility may interact to shape individual trajectories of cognitive decline. Understanding how insulin signaling, metabolic disturbances, and APOE genotype converge may provide important insights into the heterogeneity of AD and inform the development of personalized therapeutic strategies.

Future studies should also consider the clinical heterogeneity of DM2, including differences in insulin resistance, insulin secretion, disease duration, and glycemic variability, as these factors may influence the risk and progression of neurodegenerative processes associated with AD.

## 8. Conclusions and Future Perspectives

This review highlights the complex interplay between metabolic dysfunction and neurodegeneration, emphasizing the role of impaired insulin signaling as a key mechanistic link between DM2 and AD. Increasing evidence indicates that alterations in insulin signaling pathways, together with chronic inflammation, mitochondrial dysfunction, and vascular alterations, contribute to neuronal vulnerability and cognitive decline. These findings support the concept that metabolic disturbances associated with diabetes may accelerate neurodegenerative processes characteristic of AD.

At the cellular level, insulin plays essential roles in neuronal survival, synaptic plasticity, cytoskeletal organization, and energy metabolism. Disruption of insulin signaling in the brain may impair the clearance of Aβ and promote tau hyperphosphorylation, thereby facilitating the development of amyloid plaques and NFTs. In addition, genetic factors such as the APOE ε4 allele further modulate the interaction between metabolic dysfunction and neurodegeneration, highlighting the multifactorial nature of AD pathogenesis. Together, these findings support a modifier model in which metabolic dysfunction amplifies neurodegenerative vulnerability rather than acting as a primary cause of AD.

Recent advances in biomarker research have improved the ability to detect early pathological changes associated with AD. Plasma and CSF measurements of phosphorylated tau species, together with markers of metabolic dysfunction and neuroinflammation, provide valuable tools for early diagnosis and disease monitoring. At the same time, growing interest has emerged in repurposing antidiabetic drugs as potential therapeutic strategies for AD. Agents such as GLP-1RAs, SGLT2 inhibitors, and intranasal insulin have shown promising neuroprotective effects in both experimental and clinical studies, suggesting that targeting metabolic pathways may represent a viable strategy for mitigating cognitive decline. Despite growing interest in repurposing antidiabetic drugs for neurodegenerative disorders, the neuroprotective effects of these agents remain incompletely understood, and their clinical efficacy in AD will require confirmation through rigorously designed trials that address confounding, treatment duration, and CNS target engagement.

Future research should focus on clarifying the molecular mechanisms linking insulin resistance, metabolic dysregulation, and genetic susceptibility in AD. Integrating metabolic, genetic, and biomarker data may enable the identification of high-risk individuals and support the development of personalized therapeutic strategies. In addition, further clinical studies are needed to evaluate interventions aimed at restoring brain insulin signaling and improving metabolic health as potential approaches to prevent or delay neurodegeneration.

Overall, we propose that DM2 should be viewed as a disease modifier that accelerates and amplifies the progression of AD rather than a primary causative factor, acting through converging metabolic, vascular, and inflammatory pathways.

## Figures and Tables

**Table 1:** Summary of Biomarkers Related to Neurodegeneration in Diabetes Mellitus and Alzheimer’s Disease.

Biomarker	Association with DM2 and AD	Detection Method	Implications	Levels in DM2	Levels in AD	Biological Function/Pathological Role	Sample Type	Associated Clinical Stage	Therapeutic Potential/Clinical Use	References
Endothelin	Vascular damage, inflammation	Immunoassays	Indicator of endothelial dysfunction in DM2 and AD	↑	↑	Involved in vasoconstriction and vascular inflammation	Plasma/Serum	Advanced vascular stage	Vascular biomarker, possible inflammation target	[47]
Insulin	Alterations in signaling	Immunohistochemistry	Decrease in availability related to AD	↓	↓	Regulates neuronal metabolism and synaptic plasticity	Brain tissue, CSF	Early stages	Therapies aimed at improving insulin sensitivity	[48,49]
Proinsulin	Cellular stress, beta dysfunction	ELISA	Possible indicator of cellular stress and beta-cell health	↑	↑	Insulin precursor; reflects pancreatic dysfunction	Plasma	Early/progression stages	Potential early diagnostic marker	[50]
C-peptide	Influence on diabetes	Immunoassays	Linked to diabetes and suggestive of neuroprotection	Varies in DM2	Varies in AD	Modulates insulin signaling affecting neurodegeneration	Plasma	Preclinical to advanced stages	Modulates insulin signaling, potential neuroprotective.	[51]
Aβ42:Aβ40	Plaque accumulation, relation to t-tau	CSF Measurement	Associated with Aβ plaques and t-tau in AD	Altered ratio in DM2	Altered ratio in AD	Formation of amyloid plaques causing neuronal damage	CSF	Preclinical to advanced stages	Key diagnostic biomarker in AD	[52,53]
t-tau, p-tau	Neurofibrils and tangles in AD	CSF Measurement	Indicators of pathology in AD	↑	↑	Markers of neuronal damage and tau pathology	CSF	Preclinical to advanced stages	Diagnosis and monitoring of AD	[54,55]
Neuroinflammation	Inflammatory response	Inflammatory markers	Related to inflammation in DM2 and AD	↑	↑	Microglial and astroglial activation, chronic neuronal damage	Plasma, CSF	Early stages	Explored anti-inflammatory targets	[56]
GLP1-RA	GLP-1 receptor agonist	Clinical trials	Potential therapeutic intervention in DM2 and AD	Varied response in DM2	Varied response in AD	Tau aggregation causing neuronal cytoskeletal damage	Brain tissue	Progressive in AD	Potential biomarker and therapeutic target	[57,58]
Hyperphosphorylated Tau	Hyperphosphorylated Tau in AD	Western blot	Linked to tau pathology in AD	↑	↑	Improves metabolism, reduces inflammation and oxidative stress	Plasma/Serum	DM2 treatment and potential AD use	Disease-modifying therapy	[59]

This table provides an overview of key biomarkers associated with the complex interplay between DM2 and AD. The biomarkers include endothelin, insulin, proinsulin, C-peptide, Aβ42, Aβ40, t-tau, p-tau, neuroinflammation, hyperphosphorylated tau, and GLP1-RA. Each biomarker is associated with specific references that highlight its relevance in the context of neurodegeneration, insulin signaling, and AD pathogenesis. An upward arrow (↑) indicates an increase in the levels of the analyzed protein, whereas a downward arrow (↓) indicates a decrease in its levels.

## Data Availability

The original contributions presented in this study are included in the article. Further inquiries can be directed to the corresponding authors.

## Abbreviations

Aβ	Amyloid-beta
AD	Alzheimer’s Disease
AGEs	Advanced Glycation End Products
AKT	Protein Kinase B
APOE	Apolipoprotein E
BBB	Blood–brain barrier
CNS	Central Nervous System
CSF	Cerebrospinal Fluid
DM2	Type 2 Diabetes Mellitus
DPP4	Dipeptidyl Peptidase-4
GLP1-RA	Glucagon-Like Peptide-1 Receptor Agonist
GLUT1 and GLUT3	Glucose Transporters
GSK-3β	Glycogen Synthase Kinase 3 beta
IDE	Insulin-Degrading Enzyme
IGF	Insulin-like Growth Factor
IL-1β	Interleukin-1 beta
IL-6	Interleukin-6
JNK	c-Jun N-terminal Kinase
MAPK	Mitogen-Activated Protein Kinase
mTOR	Mechanistic Target of Rapamycin
NFT	Neurofibrillary Tangles
PET-FDG	Positron Emission Tomography with Fluorodeoxyglucose
PI3K	Phosphoinositide 3-Kinase
PPARγ	Peroxisome Proliferator-Activated Receptor Gamma
p-tau	Phosphorylated Tau Protein
RAGEs	Receptor for AGEs
ROS	Reactive Oxygen Species
t-tau	Total Tau Protein
VEGF	Vascular Endothelial Growth Factor
